# *SMIM1* absence is associated with reduced energy expenditure and excess weight

**DOI:** 10.1016/j.medj.2024.05.015

**Published:** 2024-06-20

**Authors:** Luca Stefanucci, Camous Moslemi, Ana R. Tomé, Samuel Virtue, Guillaume Bidault, Nicholas S. Gleadall, Laura P.E. Watson, Jing E. Kwa, Frances Burden, Samantha Farrow, DBDS Genetic Consortium, Karina Banasik, Jakob Bay, Jens Kjærgaard Boldsen, Thorsten Brodersen, Søren Brunak, Kristoffer Burgdorf, Mona Ameri Chalmer, Maria Didriksen, Khoa Manh Dinh, Joseph Dowsett, Christian Erikstrup, Bjarke Feenstra, Frank Geller, Daniel Gudbjartsson, Thomas Folkmann Hansen, Lotte Hindhede, Henrik Hjalgrim, Rikke Louise Jacobsen, Gregor Jemec, Bitten Aagaard Jensen, Katrine Kaspersen, Bertram Dalskov Kjerulff, Lisette Kogelman, Margit Anita Hørup Larsen, Ioannis Louloudis, Agnete Lundgaard, Susan, Christina Mikkelsen, Ioanna Nissen, Mette Nyegaard, Sisse Rye Ostrowski, Ole Birger Pedersen, Alexander Pil Henriksen, Palle Duun Rohde, Klaus Rostgaard, Michael Schwinn, Kari Stefansson, Hreinn Stefá nsson, Erik Sørensen, Unnur Þorsteinsdóttir, Lise Wegner Thørner, Mie Topholm Bruun, Henrik Ullum, Thomas Werge, David Westergaard, Ji Chen, MAGIC, Ji Chen, Cassandra N. Spracklen, Gaëlle Marenne, Arushi Varshney, Laura J. Corbin, Jian’an Luan, Sara M. Willems, Ying Wu, Xiaoshuai Zhang, Momoko Horikoshi, Thibaud S. Boutin, Reedik Mägi, Johannes Waage, Ruifang Li-Gao, Kei Hang Katie Chan, Jie Yao, Mila D. Anasanti, Audrey Y. Chu, Annique Claringbould, Jani Heikkinen, Jaeyoung Hong, Jouke-Jan Hottenga, Shaofeng Huo, Marika A. Kaakinen, Tin Louie, Winfried März, Hortensia Moreno-Macias, Anne Ndungu, Sarah C. Nelson, Ilja M. Nolte, Kari E. North, Chelsea K. Raulerson, Debashree Ray, Rebecca Rohde, Denis Rybin, Claudia Schurmann, Xueling Sim, Loz Southam, Isobel D. Stewart, Carol A. Wang, Yujie Wang, Peitao Wu, Weihua Zhang, Tarunveer S. Ahluwalia, Emil V.R. Appel, Lawrence F. Bielak, Jennifer A. Brody, Noël P. Burtt, Claudia P. Cabrera, Brian E. Cade, Jin Fang Chai, Xiaoran Chai, Li-Ching Chang, Chien-Hsiun Chen, Brian H. Chen, Kumaraswamy Naidu Chitrala, Yen-Feng Chiu, Hugoline G. de Haan, Graciela E. Delgado, Ayse Demirkan, Qing Duan, Jorgen Engmann, Segun A. Fatumo, Javier Gayá n, Franco Giulianini, Jung Ho Gong, Stefan Gustafsson, Yang Hai, Fernando P. Hartwig, Jing He, Yoriko Heianza, Tao Huang, Alicia Huerta-Chagoya, Mi Yeong Hwang, Richard A. Jensen, Takahisa Kawaguchi, Katherine A. Kentistou, Young Jin Kim, Marcus E. Kleber, Ishminder K. Kooner, Shuiqing Lai, Leslie A. Lange, Carl D. Langefeld, Marie Lauzon, Man Li, Symen Ligthart, Jun Liu, Marie Loh, Jirong Long, Valeriya Lyssenko, Massimo Mangino, Carola Marzi, May E. Montasser, Abhishek Nag, Masahiro Nakatochi, Damia Noce, Raymond Noordam, Giorgio Pistis, Michael Preuss, Laura Raffield, Laura J. Rasmussen-Torvik, Stephen S. Rich, Neil R. Robertson, Rico Rueedi, Kathleen Ryan, Serena Sanna, Richa Saxena, Katharina E. Schraut, Bengt Sennblad, Kazuya Setoh, Albert V. Smith, Lorraine Southam, Thomas Sparsø, Rona J. Strawbridge, Fumihiko Takeuchi, Jingyi Tan, Stella Trompet, Erik van den Akker, Peter J. van der Most, Niek Verweij, Mandy Vogel, Heming Wang, Chaolong Wang, Nan Wang, Helen R. Warren, Wanqing Wen, Tom Wilsgaard, Andrew Wong, Andrew R. Wood, Tian Xie, Mohammad Hadi Zafarmand, Jing-Hua Zhao, Wei Zhao, Najaf Amin, Zorayr Arzumanyan, Arne Astrup, Stephan J.L. Bakker, Damiano Baldassarre, Marian Beekman, Richard N. Bergman, Alain Bertoni, Matthias Blüher, Lori L. Bonnycastle, Stefan R. Bornstein, Donald W. Bowden, Qiuyin Cai, Archie Campbell, Harry Campbell, Yi Cheng Chang, Eco J.C. de Geus, Abbas Dehghan, Shufa Du, Gudny Eiriksdottir, Aliki Eleni Farmaki, Mattias Frå nberg, Christian Fuchsberger, Yutang Gao, Anette P. Gjesing, Anuj Goel, Sohee Han, Catharina A. Hartman, Christian Herder, Andrew A. Hicks, Chang-Hsun Hsieh, Willa A. Hsueh, Sahoko Ichihara, Michiya Igase, M. Arfan Ikram, W. Craig Johnson, Marit E. Jørgensen, Peter K. Joshi, Rita R. Kalyani, Fouad R. Kandeel, Tomohiro Katsuya, Chiea Chuen Khor, Wieland Kiess, Ivana Kolcic, Teemu Kuulasmaa, Johanna Kuusisto, Kristi Läll, Kelvin Lam, Deborah A. Lawlor, Nanette R. Lee, Rozenn N. Lemaitre, Honglan Li, Shih-Yi Lin, Jaana Lind-ström, Allan Linneberg, Jianjun Liu, Carlos Lorenzo, Tatsuaki Matsubara, Fumihiko Matsuda, Geltrude Mingrone, Simon Mooijaart, Sanghoon Moon, Toru Nabika, Girish N. Nadkarni, Jerry L. Nadler, Mari Nelis, Matt J. Neville, Jill M. Norris, Yasumasa Ohyagi, Annette Peters, Patricia A. Peyser, Ozren Polasek, Qibin Qi, Dennis Raven, Dermot F. Reilly, Alex Reiner, Fernando Rivideneira, Kathryn Roll, Igor Rudan, Charumathi Sabanayagam, Kevin Sandow, Naveed Sattar, Annette Schürmann, Jinxiu Shi, Heather M. Stringham, Kent D. Taylor, Tanya M. Teslovich, Betina Thuesen, Paul R.H.J. Timmers, Elena Tremoli, Michael Y. Tsai, Andre Uitterlinden, Rob M. van Dam, Diana van Heemst, Astrid van Hylckama Vlieg, Jana V. Van Vliet-Ostaptchouk, Jagadish Vangipurapu, Henrik Vestergaard, Tao Wang, Ko Willems van Dijk, Tatijana Zemunik, Goncalo R. Abecasis, Linda S. Adair, Carlos Alberto Aguilar-Salinas, Marta E. Alarcón-Riquelme, Ping An, Larissa Aviles-Santa, Diane M. Becker, Lawrence J. Beilin, Sven Bergmann, Hans Bisgaard, Corri Black, Michael Boehnke, Eric Boerwinkle, Bernhard O. Böhm, Klaus Bønnelykke, D.I. Boomsma, Erwin P. Bottinger, Thomas A. Buchanan, Mickaël Canouil, Mark J. Caulfield, John C. Chambers, Daniel I. Chasman, Yii-Der Ida Chen, Ching-Yu Cheng, Francis S. Collins, Adolfo Correa, Francesco Cucca, H. Janaka de Silva, George Dedoussis, Sölve Elmståhl, Michele K. Evans, Ele Ferrannini, Luigi Ferrucci, Jose C. Florez, Paul W. Franks, Timothy M. Frayling, Philippe Froguel, Bruna Gigante, Mark O. Goodarzi, Penny Gordon-Larsen, Harald Grallert, Niels Grarup, Sameline Grimsgaard, Leif Groop, Vilmundur Gudnason, Xiuqing Guo, Anders Hamsten, Torben Hansen, Caroline Hayward, Susan R. Heckbert, Bernardo L. Horta, Wei Huang, Erik Ingelsson, Pankow S. James, Marjo-Ritta Jarvelin, Jost B. Jonas, J. Wouter Jukema, Pontiano Kaleebu, Robert Kaplan, Sharon L.R. Kardia, Norihiro Kato, Sirkka M. Keinanen-Kiukaanniemi, Bong-Jo Kim, Mika Kivimaki, Heikki A. Koistinen, Jaspal S. Kooner, Antje Körner, Peter Kovacs, Diana Kuh, Meena Kumari, Zoltan Kutalik, Markku Laakso, Timo A. Lakka, Lenore J. Launer, Karin Leander, Huaixing Li, Xu Lin, Lars Lind, Cecilia Lindgren, Simin Liu, Ruth J.F. Loos, Patrik K.E. Magnusson, Anubha Mahajan, Andres Metspalu, Dennis O. Mook-Kanamori, Trevor A. Mori, Patricia B. Munroe, Inger Njølstad, Jeffrey R. O’Connell, Albertine J. Oldehinkel, Ken K. Ong, Sandosh Padmanabhan, Colin N.A. Palmer, Nicholette D. Palmer, Oluf Pedersen, Craig E. Pennell, David J. Porteous, Peter P. Pramstaller, Michael A. Province, Bruce M. Psaty, Lu Qi, Leslie J. Raffel, Rainer Rauramaa, Susan Redline, Paul M. Ridker, Frits R. Rosendaal, Timo E. Saaristo, Manjinder Sandhu, Jouko Saramies, Neil Schneiderman, Peter Schwarz, Laura J. Scott, Elizabeth Selvin, Peter Sever, Xiao-Ou Shu, P. Eline Slagboom, Kerrin S. Small, Blair H. Smith, Harold Snieder, Tamar Sofer, Thorkild I.A. Sørensen, Tim D. Spector, Alice Stanton, Claire J. Steves, Michael Stumvoll, Liang Sun, Yasuharu Tabara, E. Shyong Tai, Nicholas J. Timpson, Anke Tönjes, Jaakko Tuomilehto, Teresa Tusie, Matti Uusitupa, Pim van der Harst, Cornelia van Duijn, Veronique Vitart, Peter Vollenweider, Tanja G.M. Vrijkotte, Lynne E. Wagen-knecht, Mark Walker, Ya X. Wang, Nick J. Wareham, Richard M. Watanabe, Hugh Watkins, Wen B. Wei, Ananda R. Wickremasinghe, Gonneke Willemsen, James F. Wilson, Tien-Yin Wong, Jer-Yuarn Wu, Anny H. Xiang, Lisa R. Yanek, Loïc Yengo, Mit-suhiro Yokota, Eleftheria Zeggini, Wei Zheng, Alan B. Zonderman, Jerome I. Rotter, Anna L. Gloyn, Mark I. McCarthy, Josée Dupuis, James B. Meigs, Robert A. Scott, Inga Prokopenko, Aaron Leong, Ching-Ti Liu, Stephen C.J. Parker, Karen L. Mohlke, Claudia Langenberg, Eleanor Wheeler, Andrew P. Morris, Inês Barroso, The members of the Million Veteran Program contributors, Sumitra Muralidhar, Jennifer Moser, Jennifer E. Deen, J. Michael Gaziano, Sumitra Muralidhar, Jean Beckham, Kyong-Mi Chang, Philip S. Tsao, Shiuh-Wen Luoh, Juan P. Casas, Lori Churby, Stacey B. Whitbourne, Jessica V. Brewer, Mary T. Brophy, Luis E. Selva, Shahpoor (Alex) Shayan, Kelly Cho, Saiju Pyarajan, Philip S. Tsao, Scott L. DuVall, Todd Connor, Brady Stephens, Peter Wilson, Rachel McArdle, Louis Dellitalia, Kristin Mattocks, John Harley, Jeffrey Whittle, Frank Jacono, Jean Beckham, John Wells, Salvador Gutierrez, Kathrina Alexander, Kimberly Hammer, James Norton, Gerardo Villareal, Scott Kinlay, Junzhe Xu, Mark Hamner, Roy Mathew, Sujata Bhushan, Pran Iruvanti, Michael Godschalk, Zuhair Ballas, River Smith, Stephen Mastorides, Jona-than Moorman, Saib Gappy, Jon Klein, Nora Ratcliffe, Ana Palacio, Olaoluwa Oku-saga, Maureen Murdoch, Peruvemba Sriram, Shing Shing Yeh, Neeraj Tandon, Darshana Jhala, Samuel Aguayo, David Cohen, Satish Sharma, Suthat Liangpunsakul, Kris Ann Oursler, Mary Whooley, Sunil Ahuja, Joseph Constans, Paul Meyer, Jennifer Greco, Michael Rauchman, Richard Servatius, Melinda Gaddy, Agnes Wallbom, Timothy Morgan, Todd Stapley, Peter Liang, Daryl Fujii, Philip Tsao, Patrick Strollo, Edward Boyko, Jessica Walsh, Samir Gupta, Mostaqul Huq, Joseph Fayad, Adriana Hung, Jack Lichy, Robin Hurley, Brooks Robey, Prakash Balasubramanian, Urmo Võsa, Keith Burling, Lindsay Walker, John Ord, Peter Barker, James Warner, Amy Frary, Karola Renhstrom, Sofie E. Ashford, Jo Piper, Gail Biggs, Wendy N. Erber, Gary J. Hoffman, Nadia Schoenmakers, Christian Erikstrup, Klaus Rieneck, Morten H. Dziegiel, Henrik Ullum, Vian Azzu, Michele Vacca, Hugo Javier Aparicio, Qin Hui, Kelly Cho, Yan V. Sun, Peter W. Wilson, Omer A. Bayraktar, Antonio Vidal-Puig, Sisse R. Ostrowski, William J. Astle, Martin L. Olsson, Jill R. Storry, Ole B. Pedersen, Willem H. Ouwehand, Krishna Chatterjee, Dragana Vuckovic, Mattia Frontini

**Affiliations:** 1 Department of Haematology, University of Cambridge, Cambridge Biomedical Campus, Cambridge, UK; 2 National Health Service (NHS) Blood and Transplant, Cambridge Biomedical Campus, Cambridge, UK; 3 British Heart Foundation, Cambridge Centre for Research Excellence, University of Cambridge, Cambridge Biomedical Campus, Cambridge, UK; 4 Department of Clinical Immunology, Zealand University Hospital ( Roskilde University), Køge, Denmark; 5 Wellcome-MRC Institute of Metabolic Science, University of Cambridge, Cambridge, UK; 6 University of Cambridge Metabolic Research Laboratories, Institute of Metabolic Science, MDU MRC, Addenbrooke’s Hospital, Cambridge, UK; 7 NIHR Cambridge Clinical Research Facility, Cambridge University Hospitals, Cambridge Biomedical Campus, Cambridge, UK; 8 Wellcome Sanger Institute, Wellcome Genome Campus, Hinxton, Cambridge, UK; 9 Department of Clinical and Biomedical Sciences, University of Exeter Medical School, Faculty of Health and Life Sciences RILD Building, Barrack Road, Exeter, UK; 10 Estonian Genome Centre, Institute of Genomics, University of Tartu, Tartu, Estonia; 11 NIHR Cambridge Biomedical Research Centre Core Biochemical Assay Laboratory, Cambridge University Hospitals NHS Foundation Trust, Cambridge, UK; 12 NIHR National BioResource, Cambridge University Hospitals NHS Foundation, Cambridge Biomedical Campus, Cambridge, UK; 13 Discipline of Pathology and Laboratory Science, School of Biomedical Sciences, The University of Western Australia, Perth, WA, Australia; 14 Discipline of Pathology and Laboratory Medicine, Medical School, The University of Western Australia, Perth, WA, Australia; 15 Department of Clinical Immunology, Aarhus University Hospital, Aarhus University, Aarhus, Denmark; 16 Department of Clinical Medicine, Aarhus University, Aarhus, Denmark; 17 Department of Clinical Immunology, Rigshospitalet, University of Copenhagen, Copenhagen, Denmark; 18 Department of Clinical Medicine, University of Copenhagen, Copenhagen, Denmark; 19 Statens Serum Institut, Copenhagen, Denmark; 20 Department of Gastroenterology, Norfolk & Norwich University Hospitals NHS Foundation Trust, Norwich, UK; 21 Interdisciplinary Department of Medicine, Università degli Studi di Bari “Aldo Moro”, Bari, Italy; 22 Roger Williams Institute of Hepatology, London, UK; 23 Department of Neurology, Boston University School of Medicine, Boston, MA, USA; 24 Atlanta VA Medical Center, Decatur, GA, USA; 25 Department of Epidemiology, Emory University Rollins School of Public Health, Atlanta, GA, USA; 26 Massachusetts Veterans Epidemiology Research and Information Center (MAVERIC), VA Boston Healthcare System, Boston, MA, USA; 27 Department of Medicine, Brigham and Women’s Hospital, Harvard Medical School, Boston, MA, USA; 28 Emory University Schools of Medicine and Public Health, Atlanta, GA, USA; 29 Centro de Innvestigacion Principe Felipe, Valencia, Spain; 30 MRC Biostatistics Unit, East Forvie Building, Cambridge Biomedical Campus, University of Cambridge, Cambridge, UK; 31 Clinical Immunology and Transfusion Medicine, Office for Medical Services, Region Skåne, Lund, Sweden; 32 Department of Laboratory Medicine, Division of Hematology and Transfusion Medicine, Lund University, Lund, Sweden; 33 Department of Haematology, Cambridge University Hospitals NHS Trust, CB2 0QQ Cambridge, UK; 34 Department of Haematology, University College London Hospitals NHS Trust, NW1 2BU London, UK; 35 Department of Epidemiology and Biostatistics, School of Public Health, Faculty of Medicine, Imperial College London, London, UK

## Abstract

**Background:**

Obesity rates have nearly tripled in the past 50 years, and by 2030 more than 1 billion individuals worldwide are projected to be obese. This creates a significant economic strain due to the associated non-communicable diseases. The root cause is an energy expenditure imbalance, owing to an interplay of lifestyle, environmental, and genetic factors. Obesity has a polygenic genetic architecture; however, single genetic variants with large effect size are etiological in a minority of cases. These variants allowed the discovery of novel genes and biology relevant to weight regulation and ultimately led to the development of novel specific treatments.

**Methods:**

We used a case-control approach to determine metabolic differences between individuals homozygous for a loss-of-function genetic variant in the small integral membrane protein 1 (*SMIM1*) and the general population, leveraging data from five cohorts. Metabolic characterization of *SMIM1*^−/−^ individuals was performed using plasma biochemistry, calorimetric chamber, and DXA scan.

**Findings:**

We found that individuals homozygous for a loss-of-function genetic variant in *SMIM1* gene, underlying the blood group Vel, display excess body weight, dyslipidemia, altered leptin to adiponectin ratio, increased liver enzymes, and lower thyroid hormone levels. This was accompanied by a reduction in resting energy expenditure.

**Conclusion:**

This research identified a novel genetic predisposition to being overweight or obese. It highlights the need to investigate the genetic causes of obesity to select the most appropriate treatment given the large cost disparity between them.

**Funding:**

This work was funded by the National Institute of Health Research, British Heart Foundation, and NHS Blood and Transplant.

## Graphical abstract

**Figure:**
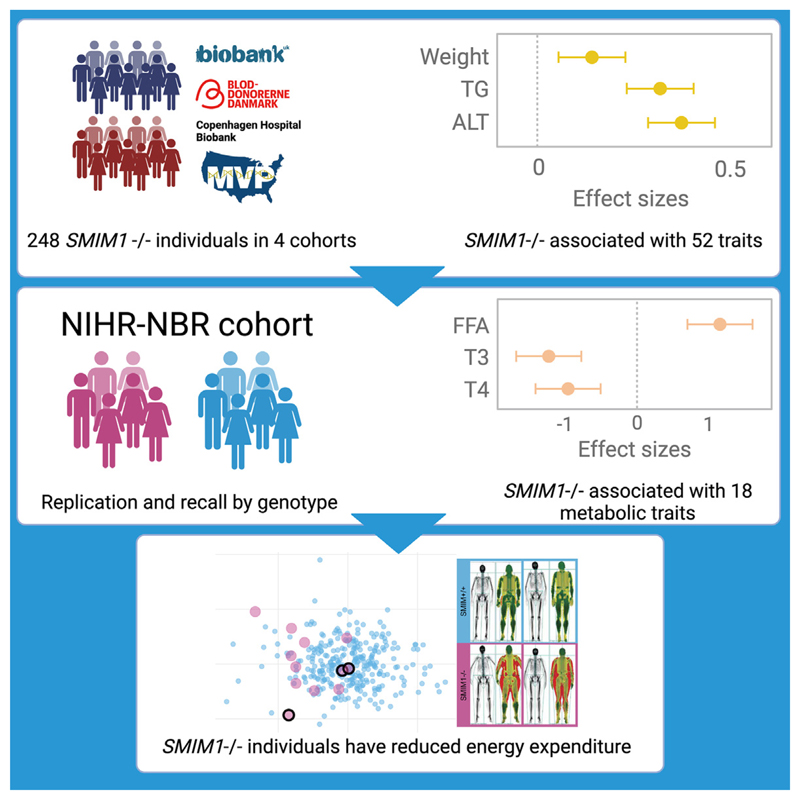
Stefanucci et al. show that a 17-bp loss-of-function variant in the open reading frame of *SMIM1* is associated with excess weight and other traits (dyslipidemia and insulin resistance) resembling metabolic syndrome. This is due to reduced energy expenditure, a major risk factor for obesity.

## Introduction

In 2013, we described a 17-bp deletion in *SMIM1* (rs566629828) that, in homozygosity, results in the absence of this protein from all tissues (hereafter *SMIM1*^−/−^) and underlies Vel-negative blood group (Figure S1).^1,2^ Over the last decade, this variant has been associated with several blood traits^3^ and, because other blood groups have been shown to be associated with different pathologies,^4–6^ we explored the impact of the absence of *SMIM1* on human health leveraging meticulously characterized phenotypic population biobanks. We analyzed UK Biobank (UKB) data to determine if the loss-of-function (LoF) variant in *SMIM1* was associated with any additional traits other than the known blood ones. In the 488,376 participants, we identified 104 individuals with *SMIM1*^−/−^ genotype, 90 being unrelated and of European ancestry^7^ (46 females and 44 males; STAR Methods, Figure S2, and Table S1), corroborating the previously reported minor allele frequency (MAF = 0.0147) for rs566629828 deletion in this ancestry^1^ and thus estimating the number of *SMIM1*^−/−^ individuals at around 200,000 worldwide. The 17-bp deletion is in high linkage disequilibrium (D^0^ 0.98) with the major (A) allele of rs1175550 (MAF = 0.78), indicating that the deletion arose on the A allele. rs1175550 is a strong sentinel expression quantitative trait locus (eQTL) for *SMIM1* in the blood^8^ (www.eqtlgen.org) and associated with red cell traits independently of rs566629828 (Figure S1).

## Results

**Figure 1:**
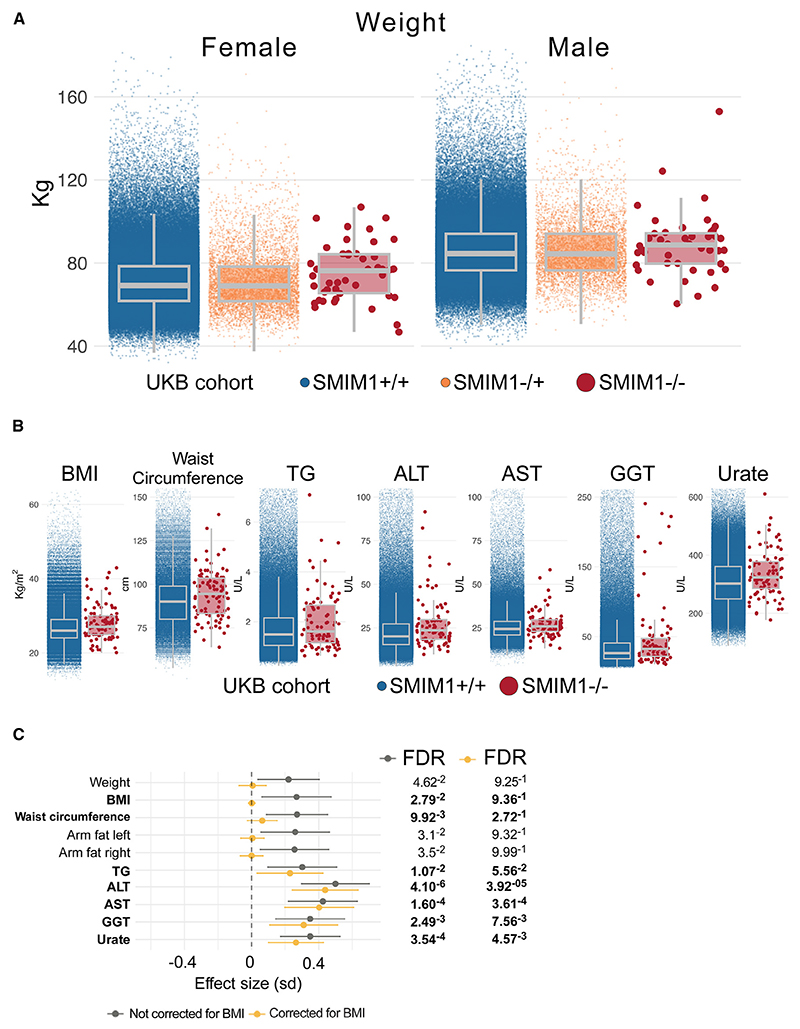
Differences between *SMIM1*^+/+^ and *SMIM1*^−/−^ individuals in the UKB cohort (A) Boxplots for UKB participants’ weight (kg) grouped according to their genotype. Sex-stratified data are shown for the three genotype groups, with females on the left and males on the right, respectively. Boxplot whiskers indicate the 95% confidence interval. (B) Boxplots for BMI, waist circumference, and levels of triglycerides (TG), alanine aminotransferase (ALT), aspartate transaminase (AST), gamma-glutamyl transferase (GGT), and urate. Boxplot whiskers indicate the 95% confidence interval. (C) Forest plot illustrating the effect size ($\hat{\beta }$ percentage of standard deviation) of *SMIM1*^+/+^ (blue) versus *SMIM1*^−/−^ (red) for each trait. Bold characters highlight the measurements that are shown in (B). Effect sizes corrected for BMI are shown in yellow, and the non-corrected ones are in dark gray; *β* is represented by the dots and the 95% confidence intervals by the horizontal lines.

We found that *SMIM1*^−/−^ participants have excess weight (linear regression, Figures 1A and S2; Table S1). This analysis indicated an autosomal recessive effect; therefore, we considered only *SMIM1*^+/+^ and *SMIM1*^−/−^ individuals for the subsequent analyses. *SMIM1*^−/−^ showed further association with body mass index (BMI) $\left(\hat{\beta }=0.27\;\text{FDR}=2.79\text{e}-2\right)$, waist circumference $\left(\hat{\beta }=0.27\;\text{FDR}\;=9.92\text{e}-3\right)$, and both arms fat mass (left: $\hat{\beta }=0.26\;\text{FDR}=3.18\text{e}-2$ right: $\hat{\beta }=0.26\;\text{FDR}=3.5\text{e}-2$ Figures 1B and 1C; Table S1). For weight, these differences equate to an average extra 4.6 kg in females and 2.4 kg in males (Table S1). In the UKB cohort, 26 out of the 90 *SMIM1*^−/−^ individuals (28.8%; 15 females and 11 males), have a BMI > 30 kg/m^2^,a higher percentage than the rest of the cohort (Fisher’s exact test odds ratio [OR] = 1.27; *p* = 1.8e−1). Analysis of UKB plasma biochemistry assay results showed that *SMIM1*^−/−^ participants had greater levels of triglycerides ($\hat{\beta }=0.3\;\text{FDR}=1.07\text{e}-2$ Figures 1B and 1C). Furthermore, they exhibited greater average levels of liver enzymes with $\hat{\beta }$ for alanine and aspartate aminotransferase of 0.50 and 0.43, and for gamma-glutamyl transferase of 0.35 (FDR = 4.10e−06, 1.60e−04, and 2.49e−03, respectively), as well as increased urate levels ($\hat{\beta }=0.35,\;\text{FDR}\;=3.54\text{e}-04$ Figures 1B and 1C; Table S1). Adjusting for the effect of BMI, removed the associations with body composition features, indicating that the higher BMI was responsible for these associations. In contrast, the associations with triglycerides, liver enzymes, and urate levels were only attenuated (Figure 1C; Table S1), suggesting that these effects were not solely dependent on BMI. Interestingly, we also identified sex-specific effects. *SMIM1*^−/−^ female UKB participants exhibit greater average fat-free mass in arms and legs (right arm $\hat{\beta }=0.39,\text{FDR}=2.39\text{e}-02$ right leg $\hat{\beta }=0.33$, FDR = 6.01e−02; Table S1) and lower average sex hormone binding globulin (SHBG) levels $\left(\hat{\beta }=-0.41,\;\text{FDR}\;=2.93\text{e}-2\right)$. Additional sex-specific differences were noted and are presented in Table S1. Importantly, (1) none of the above associations were detected in carriers of the 17-bp deletion in *SMIM1*, (2) none of the above associations were detected for the common eQTL variant rs1175550, suggesting that the metabolic differences were unlikely to be mediated by rs1175550-associated variation in the expression of *SMIM1* in red cells,^8^ (3) even when we observed differences between *SMIM1*^*+/+*^ and *SMIM1*^−*/*−^ individuals, the mean values for the two groups were within the normal ranges for each measurement, and (4) no association was found between *SMIM1*^−/−^ and fasting glucose levels in the Meta-Analyses of Glucose and Insulin-related traits Consortium (MAGIC) results.^9^

**Figure 2:**
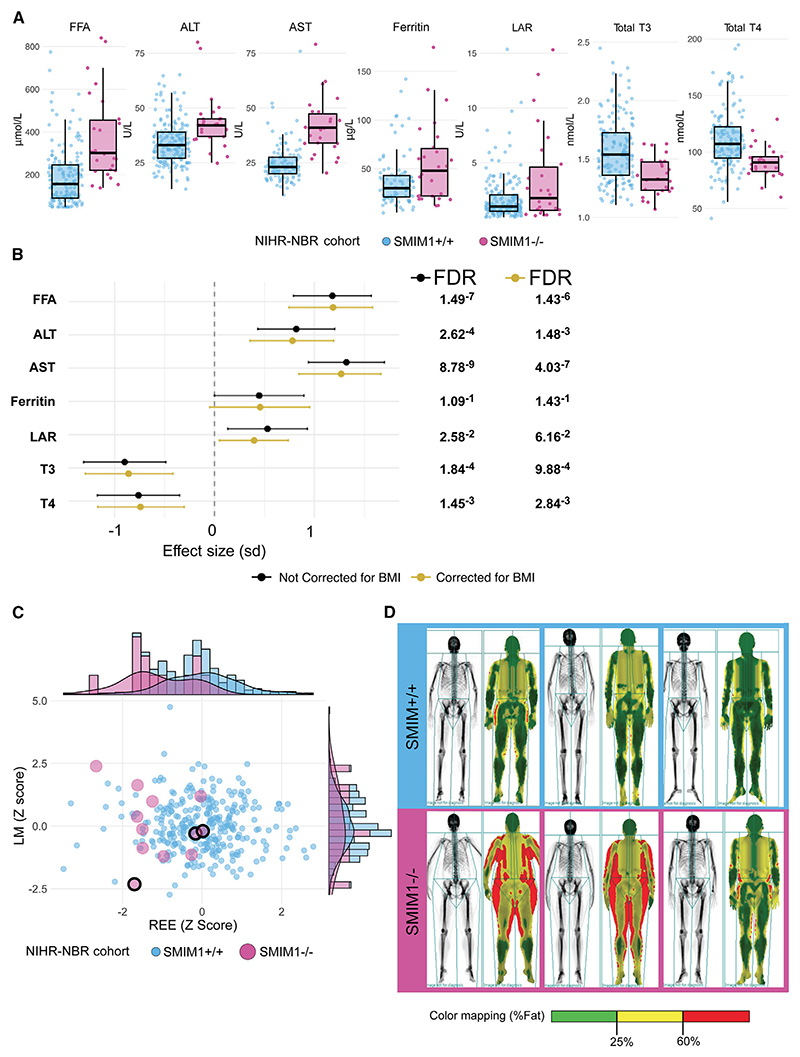
Differences between *SMIM1*^+/+^ and *SMIM1*^−/−^ individuals in the NIHR-NBR cohort and DXA body scan (A) Boxplots for free fatty acids (FFA), alanine aminotransferase (ALT), aspartate transaminase (AST), ferritin, leptin to adiponectin ratio (LAR), total triiodothyronine (T3), and total thyroxine (T4). Boxplot whiskers indicate the 95% confidence interval. (B) Forest plot illustrating the effect size ($\hat{\beta }$ percentage of standard deviation) of *SMIM1*^+/+^ versus *SMIM1*^−/−^ for each trait. Effect sizes corrected for BMI and non-corrected ones are in yellow and dark gray, respectively. *β* is represented by the dots and the 95% confidence intervals by the horizontal lines. (C) Scatterplot of *Z* scores for resting energy expenditure (REE) (x axis) and lean mass (LM) (y axis). *SMIM1*^+^ individuals, light blue; *SMIM1*^−/−^ individuals, pink. The three *SMIM1*^−/−^ individuals shown in (D) are indicated by the pink dots with a black circumference. (D) Representative DXA scans showing fat volume and distribution in three *SMIM1*^+^ participants from the control group (top row, light blue borders) and three participants from the *SMIM1*^−/−^ group (bottom row, pink borders).

To further investigate these findings, we made home visits to obtain blood samples and health data from 25 British *SMIM1*^−/−^ individuals (12 females, 13 males; not UKB participants; Figure S2) for an extensive survey of metabolism-relevant analytes, with results being compared with 180 individuals (100 females, 80 males) who carried at least one reference allele for variant rs566629828; both groups were members of the National Institute for Health and Care Research BioResource (NIHR-NBR) (all values in Table S2). We observed the same trend for *SMIM1*^−/−^ individuals to be heavier but, possibly due to the small sample size, the significance threshold was not reached (Table S3). We replicated the associations between *SMIM1*^−/−^ for increased average levels for alanine aminotransferase and aspartate transaminase, with the same order of magnitude as observed in UKB (Figure 2; Table S3). We also found associations between *SMIM1*^−/−^ and increased leptin to adiponectin ratio (LAR) ($\hat{\beta }=0.53$, FDR = 2.58e−02), and an increase in free fatty acids ($\hat{\beta }=1.18$, FDR = 1.43e−06), two indices of increased fat mass and insulin resistance (Figures 2A and 2B).^10,11^ LAR (a marker for obesity and metabolic state^12,13^) increase was determined by an increase in leptin $\left(\hat{\beta }=0.38\right)$ and a reduction in adiponectin $\left(\hat{\beta }=-0.37\right)$, albeit with *p* values slightly above the defined significance level (0.06 and 0.09, respectively).

Moreover, we found that *SMIM1*^−/−^ individuals have lower average levels of total triiodothyronine (T3) and thyroxine (T4) ($\text{T}3:\hat{\beta }=-0.86$, FDR = 9.87e-04; $\text{T4}:\hat{\beta }=-0.74$, FDR = 2.84e-03; Figures 2A and 2B), whereas the levels of thyroid-stimulating hormone (TSH), albeit skewed toward the bottom of the normal distribution, were not different (Table S3).

The above findings prompted us to invite 12 *SMIM1*^−/−^ individuals belonging to the NIHR-NBR cohort for a 2-day metabolic assessment (Figure S2). We measured the effect of the absence of *SMIM1* on resting energy expenditure (REE) (a marker of whole-body metabolic activity) by indirect calorimetry and body mass composition by dual-energy X-ray absorptiometry (DXA), using a well-established protocol (STAR Methods).^14^ These studies showed that *SMIM1*^−/−^ individuals had a lower REE adjusted for lean mass (Figures 2C and x axis; Wilcoxon rank-sum test; *p* = 2.16e-04, Table S4), while there were no differences in average lean mass compared with 310 unselected controls (Table S4). Average free T3, but not free T4, measurements were lower in the 12 *SMIM1*^−/−^ than in the control group (Tables S2 and S3). Lower circulating total thyroid hormones in SMIM1^-/-^ individuals are not due to reduction in thyroid hormone binding globulin and thyroglobulin levels are not elevated, making thyroid dyshormonogenesis an unlikely cause of their altered thyroid status (Table S2). The anthropometric differences observed in the 90 *SMIM1*^−*/*−^ UKB participants were reflected in abnormal body composition visualized by DXA scans (Figure 2D). Because of the effect on REE, T3, and T4 levels, we explored the possible involvement of *SMIM1* in the hypothalamic-pituitary-thyroid axis.^15^ To gain insight into the possible molecular mechanism(s), we analyzed the single-cell RNA sequencing data in studies that dissected the transcript levels of these tissues in multiple organisms. In the mouse hypothalamus^16^ (GSE113576), *Smim1* was expressed at low levels in mature oligodendrocytes and some, but not all, inhibitory neurons (Figure S3A). Its expression was largely non-overlapping with that of the thyrotropin-releasing hormone (Figure S3B). In the human anterior pituitary gland^17^ (GSE142653), *SMIM1* was expressed in corticotropes, gonadotropes, and somatotropes (Figure S3C), while in human thyroid organoids and mouse thyroid^18^ (GSE163818) low-level expression was detected mainly in thyrocytes and in, as yet, uncharacterized Flt1-positive cells (Figure S3C). These analyses indicate that *SMIM1* could play one or more roles in the hypothalamic-pituitary-thyroid axis.

The associations between the genotype at rs566629828 and phenotypes observed in the UKB and NIHR-NBR cohort were orthogonally validated in 73 Danish *SMIM1*^−/−^ individuals from the Danish Blood Donor Study^19^ (DBDS) (blood donor, 25 females, 18 males, and 645 controls), and the Copenhagen Hospital Biobank^20^ (CHB) (hospitalized or outpatients, 12 females,18 males, and 450 matched controls). Weight data, available only for the DBDS participants, showed, upon bootstrapping analysis (STAR Methods; controls matched by age, sex, and smoking status), consistent directionality for female *SMIM1*^−/−^ individuals. However, the low number of Danish *SMIM1*^−/−^ individuals and a less evident effect on weight in males (Figure 1B) limited the statistical power to detect differences. A meta-analysis combination of *SMIM1*^−/−^ individuals from the Million Veteran Program (MVP) and the cohorts described above yielded the same directionality of effect (Figure S4). Interestingly, 20 of the 73 (27%) *SMIM1*^−/−^ individuals in the Danish cohorts were diagnosed with lipoprotein metabolism disorders versus 13% in the controls (OR = 4.07, FDR = 2.09e−04). A review of all prescriptions in both cohorts showed greater use of statins in individuals lacking *SMIM1* versus controls (OR = 2.36, FDR = 2.22e−02; Table S5), indicating a higher number of individuals considered predisposed to cardiovascular events. An exploratory analysis of hospital episode statistics revealed an increased risk for cerebral events, with 5 cerebral bleeds and 5 thrombotic strokes in the 65 *SMIM1*^−*/*−^ UKB participants for whom hospital event statistics were available (OR = 5.53 and 3.46, FDR = 6.88e−04 and 2.32e−02, respectively; Table S6).

## Discussion

In summary, our analysis identified a novel autosomal recessive effect for a LoF deletion (i.e., rs566629828) in *SMIM1*, a protein of yet unknown function(s), up until now only known as the antigen underlying the Vel blood group. This variant is present in at least 200,000 individuals worldwide (1 in 5,000 individuals in Great Britain and higher frequency in the Scandinavian countries [this article and Storry et al.^2^] and extremely low frequency in other ancestries). We have shown that *SMIM1*^−/−^ individuals (Vel-negative blood group) exhibit a combination of metabolic features, including excess fat mass, inflammation, altered liver function, triglycerides, and altered lipoprotein metabolism. These features are due, at least in part, to reduced energy expenditure, a major risk factor in obesity.^21,22^ In the most extreme cases, these effects could lead to an increased risk of insulin resistance and metabolic syndrome onset, accompanied by an increased susceptibility to cardiovascular disease, as supported by drug prescription and electronic hospital records analyses. These indicated that *SMIM1*^−/−^ individuals have a higher likelihood of being prescribed statins and may be more prone to cerebral bleeds and thrombotic stroke. The effect on fat mass, and associated traits, is likely secondary, as suggested by its dependence on BMI, while we foresee a direct effect of *SMIM1* on dyslipidemia and liver function as these parameters continue to hold significance even after BMI correction. The associated phenotypes also show sexual dimorphism in their presentation. While the weight differences between the sexes likely reflect the different distribution of this tissue between males and females,^23^ association with traits such as urate and gamma-glutamyl transferase were driven by a stronger effect in one sex; whereas others, such as SHBG, were found only in females. Altogether, the observed metabolic phenotype, the increased risk for cardiovascular events, and the expression pattern of *SMIM1* are compatible with the fact that its absence results in a state of mild hypothyroidism.

The minor allele frequency of rs566629828 is at the lower end of common variations (MAF = 0.0147) and has one of the largest effects on weight $\left(\hat{\beta }=0.22\right)$ and BMI $\left(\hat{\beta }=0.27\right)$ reported so far, with the exception of extremely rare variants directly implicated in lipid metabolism.^24^ For comparison, genetic variants in other well-characterized genes associated with obesity, i.e., *PCSK1* and *MC4R*, have comparable effect sizes in the general population. In particular, *MC4R* became a drug target for weight control in severe forms of genetically caused obesity.^24–26^

The rapidly growing amount of genomic data available, including blood donors typed by arrays,^27^ means that more and more *SMIM1*^−*/*−^ individuals will be identified as part of the incidental findings. Those who received a test early in life should be advised to monitor their energy intake, while individuals already overweight or obese, could be treated with a levothyroxine supplementation, an extremely cost-effective option compared with the most recent recommendations for the treatment of obesity.^28,29^ While the effects have been replicated in independent cohorts, documenting central hypothyroidism with normal TSH, but low free thyroid hormone, measurements in a larger number of *SMIM1*^−/−^ individuals will be necessary, before advocating a clinical trial of levothyroxine treatment as a potential therapeutic intervention.

### Limitations of the study

The analyses we presented here are limited by the infrequency of *SMIM1*^−*/*−^ individuals. To obtain sufficient statistical power, we had to use cohorts collected with different aims and with only a partial set of overlapping measurements. Differences in cohort composition and lifestyle might confound the observed effects on metabolism, and these biases could still influence the meta-analysis results. To limit confounding effects, controls were selected within the same cohort. This is exemplified in the meta-analysis (Figure S4), where only four *SMIM1*^−/−^ individuals in the MVP cohort are female. We believe that the deep phenotype characterization of the recalled individuals and their consistency with the effect observed in the population helped to overcome this limitation. Future studies should investigate the function of this small transmembrane protein to identify the mechanisms by which it affects metabolism, as this could pave the way to novel therapeutic opportunities.^30–32^

### Star★Methods

Detailed methods are provided in the online version of this paper and include the following:

- KEY RESOURCES TABLE
- RESOURCE AVAILABILITY
  ○ Lead contact
  ○ Materials availability
  ○ Data and code availability
- EXPERIMENTAL MODEL AND STUDY PARTICIPANT DETAILS
  ○ UK Biobank (UKB) cohort
  ○ NIHR-NBR cohort
  ○ Danish Blood Donor Study (DBDS) and Copenhagen Hospital biobank (CHB) cohorts
  ○ Million Veteran Program (MVP) cohort
- METHOD DETAILS
  ○ Confirmation of rs566629828 genotype status
  ○ Metabolic characterization
  ○ Single-cell RNA-seq analyses
- QUANTIFICATION AND STATISTICAL ANALYSIS

## Star★Methods

### Key Resources Table

**Table:** 

REAGENT or RESOURCE	SOURCE	IDENTIFIER
Biological samples		
Vel-negative blood samples	NIHR Clinical Research Facility	https://www.cambridgecrf.nihr.ac.uk/
NIHR-NBR cohort	This article	https://bioresource.nihr.ac.uk/
Critical commercial assays		
Atellica IM Free Thyroxine lite reagent	Siemens Healthineers	10995589
Immulite 2000 TBG assay	Siemens Healthineers	L2KTB2
Total cholesterol	Siemens Healthineers	DF27
High-density lipoprotein	Siemens Healthineers	DF48B
Aspartate aminotransferase	Siemens Healthineers	DF41A
Alanine transaminase	Siemens Healthineers	DF 143
High-sensitivity C-reactive protein	Siemens Healthineers	RF 434
Free Fatty Acids	Sigma Aldrich (Roche)	11 383 175 001
Thyroid-stimulating hormone	DiaSorin	311211
TotalT3	DiaSorin	311311
TotalT4	DiaSorin	311411
Free T4	DiaSorin	311611
Ferritin	DiaSorin	313551
Leptin (Not a commercially available assay)	DELFIA®	https://www.perkinelmer.com/uk/lab-products-and-services/application-support-knowledgebase/delfia/delfia-products-catalog.html
Adiponectin (Not a commercially available assay)	DELFIA®	https://www.perkinelmer.com/uk/lab-products-and-services/application-support-knowledgebase/delfia/delfia-products-catalog.html
Deposited data		
NIHR-NBR cohort metabolites - raw data	This article, and Zenodo	https://zenodo.org/records/10685501
NIHR-NBR cohort resting energy expenditure - raw data	This article, and Zenodo	https://zenodo.org/records/10685501
UK Biobank	Downey et al.^34^	https://www.ukbiobank.ac.uk/
Million Veteran Program	Gaziano et al.^44^	https://www.research.va.gov/mvp/
Danish Blood Donor Study	Hansen et al.^19^	https://bloddonor.dk/
Copenhagen Hospital Biobank	Sorensen et al.^20^	https://www.regionh.dk/blodbanken/afdelingen/enheder-paa-rigshospitalet/Sider/biobank.aspx
Mouse hypothalamus	Moffitt et al.^16^	GEO: GSE113576
Human fetal pituitary	Zhang et al.^17^	GEO: GSE142653
Mouse pituitary	Cheung et al.^53^	GEO: GSE120410
Mouse pituitary	Ho et al.^54^	GEO: GSE146619
Rat pituitary	Fletcher et al.^55^	GEO: GSE132224
Mouse thyroid organoids	Romitti et al.^18^	GEO: GSE163818
Oligonucleotides		
Primer: *SMIM1* deletion Forward: ACAGCCTGGCCACCTGTCTTG Reverse: CTGCGGCAGCGTGAGGC	This article	N/A
Software and algorithms		
R 4.0.5	R Core Team	https://www.r-project.org/
Tidyverse 1.3.1	RStudio Team	https://tidyverse.tidyverse.org
meta 5.2–0	Schwarzer et al.^54^	N/A
Seurat (version 4.0.0)	Hao et al.^33^	https://satijalab.org/seurat/
biomaRt (version 2.46.3)	Durinck et al.^35^	https://bioconductor.org/packages/release/bioc/html/biomaRt.html
RNOmni	McCaw et al.^45^	https://cran.r-project.org/web/packages/RNOmni/index.html
metafor 3.0–2	Viechtbauer et al.^37^	https://github.com/wviechtb/metafor
GE Lunar iDXA - Encore version 18	GE Healthcare	210500GA
GE Lunar Prodigy - Encore version 16	GE Healthcare	N/A
Gas Exchange Measurement	GEMNutrition Ltd, Daresburysbury	17040–181
PLINK v1.9	PLINK working group	https://www.cog-genomics.org/plink/1.9/
Other		
Dimension EXL	Siemens Healthineers	https://www.siemens-healthineers.com/
Liaison XL	DiaSorin	https://int.diasorin.com/en

## Resource Availability

### Lead contact

Further information and requests for resources should be directed to and will be fulfilled by the lead contact, Mattia Frontini (m.frontini@exeter.ac.uk).

### Materials availability

This study did not generate new unique reagents.

## Experimental Model and Study Participant Details

A total of 248 *SMIM1*^−*/*−^ unrelated European individuals (105 females, 143 males) from four different cohorts, described below were included in the study. For an overview of the cohorts, see Figure S2. Details about the recall by genotype study and the individual cohorts are reported below in the relevant sections.

### UK Biobank (UKB) cohort

The UKB analyses have been conducted under application number 13745. This cohort consists of 502,682 participants, aged between 40 and 69 years of age on enrollment, recruited at 22 assessment centers across the UK between 2006 and 2010.^38,39^ DNA samples were taken from participants and genotyped using the UK Biobank Axiom Array on the GeneTitan (Affymetrix, Santa Clara, CA). Genotype calling and quality control of the UKB dataset have been extensively documented elsewhere.^7^

The UKB Axiom Array contains DNA probes for direct genotyping of the variant underlying the 17-bp deletion in *SMIM1* (NC_000001.11:g.3775437_3775453del, rs566629828). The specific DNA probes used (Probeset ID: AX-86577342, Variant ID: Affx-80267180) for detection of the deletion have shown high specificity and the rare variant can be reliably called.^27^ For this study, only directly measured genotypes for variant rs566629828 were used to identify UKB participants homozygous for the 17-bp deletion in *SMIM1*, as opposed to imputed genotypes. Additionally, manual inspection of genotype call plots for the deletion probeset (AX-86577342) was performed for each of the 106 genotyping batches of 4,700 UKB samples. rs566629828 is in Hardy-Weinberg equilibrium (*X*^*2*^ test, *p-value* = 0.92) with an allele distribution, in UKB, of +/+ = 396559, +/−= 11849, −/−= 90 and a theoretical expected distribution of +/+ = 396558, +/−= 11852, −/−= 89. The linkage disequilibrium score D^0^ value between variants rs1175550 and rs566629828 was calculated using PLINK software.^40^ The clinical phenotypes have been defined according to the Hospital Episode Statistics (HES) recorded for the majority of UKB participants. The list of ICD-10 codes and fields used to select the cases and traits is in Table S7.

### NIHR-NBR cohort

All NIHR-NBR participants for this study were recruited under approval 12/EE/0040 by the Research Ethics Committee East of England. Initially, the *SMIM1*^−*/*−^ individuals were identified by the testing approach outlined in an earlier publication.^1^ In short, the red cells of a small portion of the 1.4 million donations collected annually are tested by haemagglutination for the presence of the Vel blood group antigen with a specific typing reagent. Individuals with a negative result and part of the NIHR-NBR underwent a confirmatory polymerase chain reaction test for the 17-bp deletion. Of these, 25 participated in this study by providing samples and relevant health information obtained during a home visit. Twelve of the 25 attended the NIHR Clinical Research Facility at Cambridge University Hospitals (Cambridge, UK) for a 2-day metabolic assessment, no exclusion criteria were applied. The measurement results of the *SMIM1*^−*/*−^ individuals were compared with the results obtained for 180 NIHR-NBR participants (100 females, 80 males) with a reference/reference or reference/alternate genotype for variant rs566629828 as previously determined by whole-genome sequencing.^41^

### Danish Blood Donor Study (DBDS) and Copenhagen Hospital biobank (CHB) cohorts

The CHB participants were recruited under the NVK-1708829, P-2019-93 approval and the DBDS participants were recruited under the 1-10-72-95-13, NVK-1700407, P-2019-99 approval. The CHB participants have been enrolled at the Copenhagen University Hospital and general hospitals in the region of greater Copenhagen. Inclusion in the study is limited to patients attending to these hospitals and from whom a blood sample is drawn for ABO and D grouping and/or red cell antibody screening. The cohort is therefore strongly skewed toward patients with medical conditions associated with a high likelihood of requiring transfusion (e.g., surgery, chemotherapy and pregnancy).^20^ The DBDS cohort of Danish blood donors is in demographics similar to the NIHR-NBR cohort from whom 25 British *SMIM1*^−/−^ individuals were drawn.^19,42^

The DNA samples from the 90,000 DBDS and 90,700 CHB participants were genotyped at deCODE Genetics (Reykjavik, Iceland) using the Infinium Global Screening Array (Probeset ID: GSA-24v1-0_C2, v1.0). Imputation of the 17-bp deletion rs566629828 was performed by deCODE Genetics using their North European sequencing panel of 15,576 individuals (including 8,429 Danes) as reference. Based on these two imputed datasets, 49 and 34 individuals were identified in DBDS and CHB, with a high likelihood of being homozygous for the 17-bp deletion in *SMIM1*, respectively. The DBDS and CHB participant and genotyping data are linked to the Danish Laboratory Database (DLD), the Danish National Patient Registry^43^ (NPR) and the Danish Prescription Database^43^ (DPD). These linked databases were used for the association analysis performed for this study.

### Million Veteran Program (MVP) cohort

The design of MVP has been previously described.^44^ Veterans were recruited from over 60 Veterans Health Administration medical centers nationwide since 2011. A unique feature of MVP is the linkage of a large biobank to an extensive, national, database from 2003 onward that integrates multiple elements such as diagnosis codes, procedure codes, laboratory values, and imaging reports, which permits detailed phenotyping of this large cohort. MVP has received ethical and study protocol approval by the Veterans Affairs Central Institutional Review Board in accordance with the principles outlined in the Declaration of Helsinki.

DNA extracted from participants’ blood was genotyped using a customized Affymetrix Axiom biobank array, the MVP 1.0 Genotyping Array. The array was enriched for both common and rare genetic variants of clinical significance in different ethnic backgrounds. Quality-control procedures used to assign ancestry, remove low-quality samples and variants, and perform genotype imputation were previously described.^45^ We excluded: duplicate samples, samples with more heterozygosity than expected, an excess (>2.5%) of missing genotype calls, or discordance between genetically inferred sex and phenotypic gender.^45^ In addition, one individual from each pair of related individuals (more than second-degree relatedness as measured by the KING software were removed.^46^ SNP rs566629828 (*SMIM1*) was directly genotyped on the MVP array. The MVP participants were assigned to mutually exclusive racial/ethnic groups using HARE (Harmonized Ancestry and Race/Ethnicity), a machine-learning algorithm that integrates genetically inferred ancestry with self-identified race/ethnicity.^47^ The present study included non-Hispanic European Americans with both genotypic and phenotypic data for genetic association analyses. The details of the genetic association study of BMI in the MVP were previously described.^48^

## Method Details

### Confirmation of rs566629828 genotype status

Considering the limited accuracy of imputation to determine the genotype of low-frequency variants, and particularly of indels,^49^ the genotype at rs566629828 was confirmed by an orthogonal test^2^ using DNA extracted from 49 DBDS and 34 CHB blood samples, which were retrieved from the respective sample repositories. In short, DNA was amplified by primers flanking the 17-bp deletion in *SMIM1* exon 3 (Figure S1). The amplicons were resolved by agarose gel electrophoresis and visual inspection of the amplicon length (reference and alternate alleles being 178bp and 161bp in length, respectively). Discordant results between the genotype inferred by imputation and the PCR-genotyping test results were observed for 10 DNA samples (DBDS, *n* = 6; CHB, *n* = 4). These ambiguities were resolved by Sanger sequencing of the *SMIM1* coding exons 3 and 4 confirming that all 10 discordances were caused by erroneous imputation results. All together 43 and 30 confirmed *SMIM1*^−*/*−^ individuals were identified in the DBDS and CHB cohorts, respectively (Figure S2). Controls are drawn from the same cohorts in a ratio of 15:1, gender and age-matched (DBDS, *n* = 645; CHB, *n* = 450). The genotype of the controls was imputed and it was either reference/reference or reference/alternate for the variant rs566629828.

### Metabolic characterization

The Cambridge Central East of England Research Ethics Committee approved the study protocol for participants’ metabolic characterization (06/Q0108/84). NIHR-NBR participants were asked to refrain from exercise, consume alcohol and caffeine for 24 h before arrival. Each of the 12 participants arrived at the NIHR Clinical Research Facility at Cambridge University Hospitals at 14:00 h on day 0 and remained until noon on day 1. Resting energy expenditure was measured upon waking after an overnight fast by indirect calorimetry (GEM Nutrition) using a ventilated hood. Gas analysis exchange measurements were converted into energy equivalents using calculations by Elia and Livesey.^50^ The procedure and precision values of the indirect calorimetry method have been previously described.^51^ Whole-body fat, lean and bone mass body composition measurements were performed by Dual Energy X-ray Absorptiometry (DXA). For the volunteers homozygous for the 17-bp deletion in *SMIM1*, GE Lunar iDXA (Encore version 18) was used for fat mass, lean mass and bone mineral content (BMC) measurements. For the controls, there was a combination of GE Lunar iDXA measurements and GE Lunar Prodigy measurements (Encore version 16). Therefore, all relevant measurements were converted by cross-calibration equations^52^ to comparable iDXA values before collating and using regression modeling. Lean mass and resting energy expenditure Z scores were derived by multiple regression modeling.^14^ The coefficients were updated in line with an upgrade in DXA scanner (resting energy expenditure (kJ/min) = age; −0.015, fat mass (kg); 0.019, lean mass (kg); 0.063, intercept; 1.580, lean mass (kg) = gender (0; male, 1; female); −6.272, height2 (m2); 6.684, bone mass (kg); 10.458, fat mass (kg); 0.166, intercept; 0.888).

### Single-cell RNA-seq analyses

We analyzed single-cell RNA-sequencing data from the following sources:

Mouse hypothalamus (GSE113576)^16^
Human fetal pituitary (GSE142653)^17^ human in Figure S3E.
Mouse pituitary (GSE120410)^53^ mouse in Figure S3E.
Mouse pituitary (GSE146619)^54^ mouse in Figure S3E.
Rat pituitary (GSE132224)^55^ rat in Figure S3E.
Mouse thyroid organoids (GSE163818)^18^

Normalization, visualization, and standard processing of datasets was done through Seurat.^34^ For label transfer of mouse and rat pituitary datasets from the human pituitary reference: mouse and rat genes were first converted to their human homologs (as obtained via BioMart^35^), and ambiguously annotated genes were filtered out, prior to cross-species integration.

## Quantification And Statistical Analysis

Linear regression was performed to estimate the effect of continuous variables. The statistical model used as covariates age, sex and BMI. Sex and BMI were removed from the equation when considering sex-stratified data or the effect of the variant on BMI, respectively. Similarly, for categorical variables, the explanatory effect of variant rs566629828 was estimated by logistic regression. For continuous traits, inverse normal transformation (R package RNOmni) was adopted to normalize the measurements.^36^ In the Danish cohorts, the logistic regression analysis was performed with the response variable defined as the presence of an abnormal Nomenclature for Properties and Units (NPU)-code measurement in the DLD dataset, presence of a given ICD10/ICD8 record in the NPR dataset, or presence of a specific prescription in the DPD dataset. The explaining variables used were variant rs566629828 genotype, age of the individuals, genetically inferred sex of the individuals (unless cohort was sex-stratified), and in case of mixed cohort analysis, the cohort of a given individual (DBDS/CHB). Since weight data does not follow a normal distribution, a Wilcoxon signed-rank test was used to assess differences in mean weight-based on variant rs566629828 genotype after sex stratification. Bootstrapping was used to assess directionality in mean weights based on the rs566629828 genotype. For each *SMIM1*^−/−^ DBDS case, 100 alternate age, sex and smoking status-matched control groups were selected at random. The mean weight of each of these 100 alternate control groups was compared to the case group’s mean weight. Directionality of the difference in mean weights was then assessed for each sex separately. Statistical tests have *p* values corrected with Benjamini–Hochberg procedure with alpha set at 0.05. The meta-analysis pooling across the cohorts was performed with inverse variance and the R package meta (version 5.2–0).

## Supplementary Material

Supplemental information

## Data Availability

Participants’ phenotypes and *SMIM1* locus genotypes are accessible via the relevant cohort environments: UK Biobank (https://www.ukbiobank.ac.uk/) and MVP (https://www.mvp.va.gov/pwa/discover-mvp-data). Access to these cohorts requires an active project application. All the data generated for this study are available in an anonymized version in supplementary tables or in the Zenodo repository at: https://zenodo.org/records/10685501. The code used to analyze the cohorts is available at https://github.com/stefanucci-luca/vel_ko_analysis. Any additional information required to reanalyze the data reported in this paper is available from the lead contact upon request.

## Highlights

*SMIM1*^−/−^ individuals are heavier compared with their *SMIM1*^+/+^ counterparts

*SMIM1*^−/−^ individuals have dyslipidemia and a higher likelihood of being on statins

*SMIM1*^−/−^ individuals exhibit traits that resemble metabolic syndrome onset

Reduced energy expenditure due to mild hypothyroidism could be the underlying cause

## Context and Significance

Stefanucci et al. investigate the effects on human health of a 17-nucleotide deletion in the *SMIM1* gene, known so far for encoding the antigen of the Vel blood group. In the article, the authors show, using large population cohorts, such as the UK Biobank, that the absence of *SMIM1* is linked to excess weight, dyslipidemia, and related traits. The metabolic characterization of *SMIM1*^−/−^ individuals points toward reduced energy expenditure as the main contributor to weight gain. Further research to understand the molecular mechanisms behind *SMIM1-*associated phenotypes might unlock new treatments for obesity and related conditions. This work highlights the importance of genetic and molecular insights in tackling metabolic disorders and improving public health.
