# Supplementary material for: SMIM1 absence is associated with reduced energy expenditure and excess weight
Source: Med. Author manuscript; Available in PMC 2025 Feb 24. (PMC7617389; doi:10.1016/j.medj.2024.05.015)
Supplement: Supplemental information [file EMS202150-supplement-Supplemental_information.zip › DataS1/SF files/SF4.pdf]

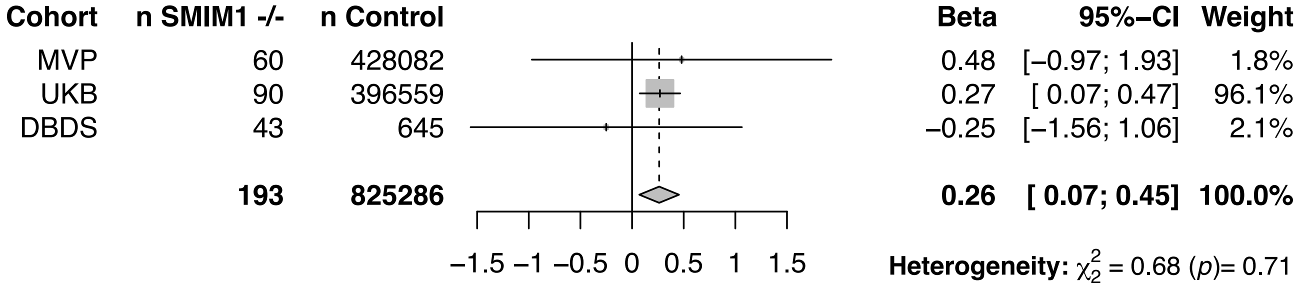

SF4 | Forest plot displaying SMIM1 -/- meta-analysis results across population scale cohorts. Each row corresponds to a study and reports the number of participants used in the analysis and beta estimate. The last row, with the diamond-shaped point represents the pooled effect of the different cohorts. The “weight” column indicates the percentage contribution of each study to the meta-analysis outcome. Heterogeneity across the cohorts has been measured with the chi-squared test. The P value heterogeneity does not highlight any heterogeneity problem across the cohorts. MVP = Million Veteran Program; UKB = UK Biobank; DBDS = Danish Blood Donor Study; CI = confidence interval.
