# Supplementary material for: SMIM1 absence is associated with reduced energy expenditure and excess weight
Source: Med. Author manuscript; Available in PMC 2025 Feb 24. (PMC7617389; doi:10.1016/j.medj.2024.05.015)
Supplement: Supplemental information [file EMS202150-supplement-Supplemental_information.zip › DataS1/SF files/SF3.pdf]

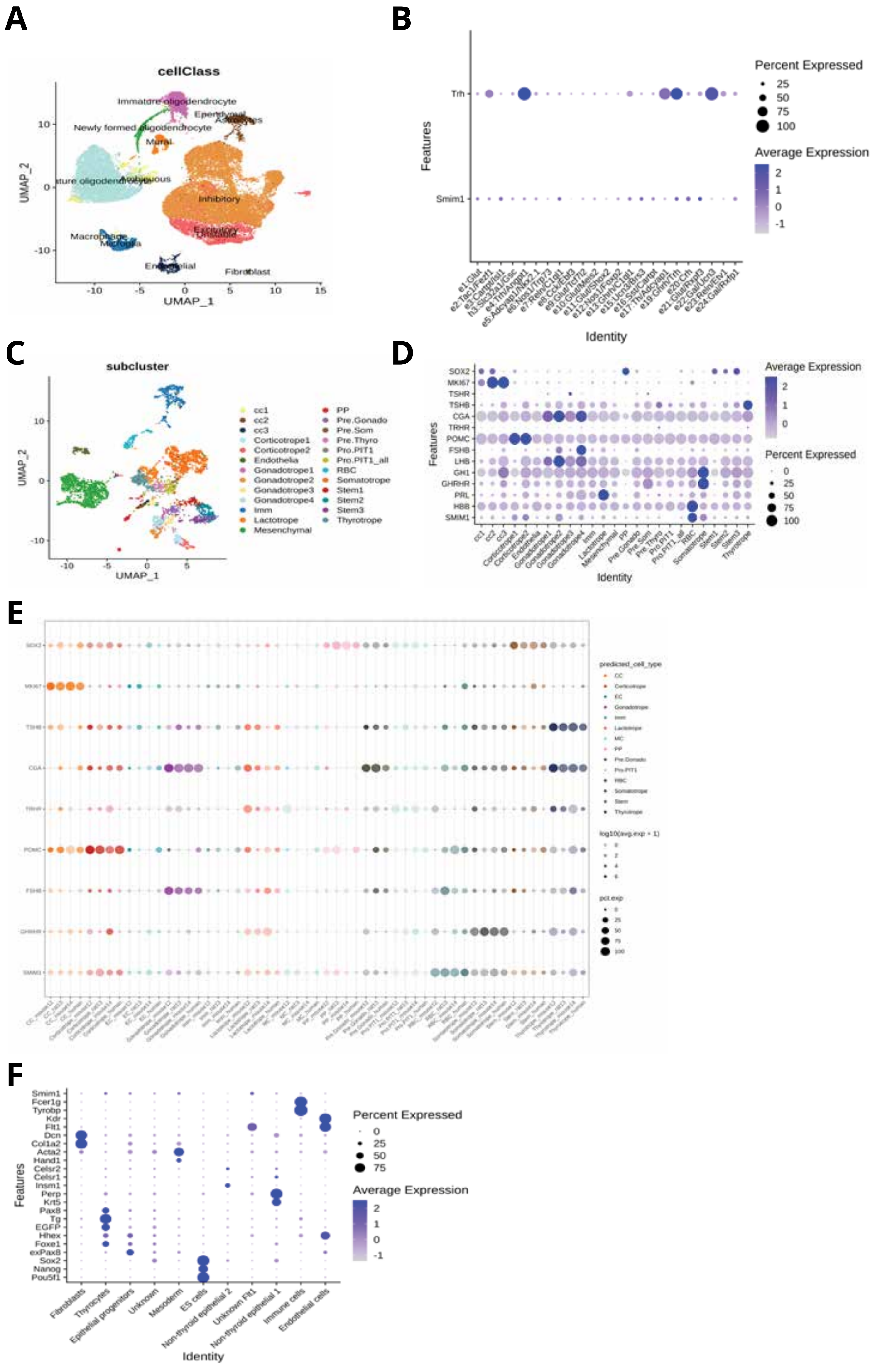

SF3 | SMIM1 expression at single-cell resolution in mammalian hypothalamus, pituitary and thyroid. (A) UMAP representation of the different cell types found in mouse hypothalamus snRNA-seq data (GSE113576). **(B)** Smim1 and Trh expression levels in the different cell types found in mouse hypothalamus. **(C)** UMAP representation of the different cell types found in human fetal pituitary scRNA-seq data (GSE142653). **(D)** SMIM1 and representative genes expression levels in the different cell types found in the human fetal pituitary. **(E)** Smim1 and representative genes expression levels in the different cell types found in rat and mouse pituitary. **(F)** Smim1 and representative genes expression levels in the different cell types found in mouse thyroid organoids (GSE163818).
