# Supplementary material for: SMIM1 absence is associated with reduced energy expenditure and excess weight
Source: Med. Author manuscript; Available in PMC 2025 Feb 24. (PMC7617389; doi:10.1016/j.medj.2024.05.015)
Supplement: Supplemental information [file EMS202150-supplement-Supplemental_information.zip › DataS1/SF files/SF2.pdf]

Supplementary Figure 2

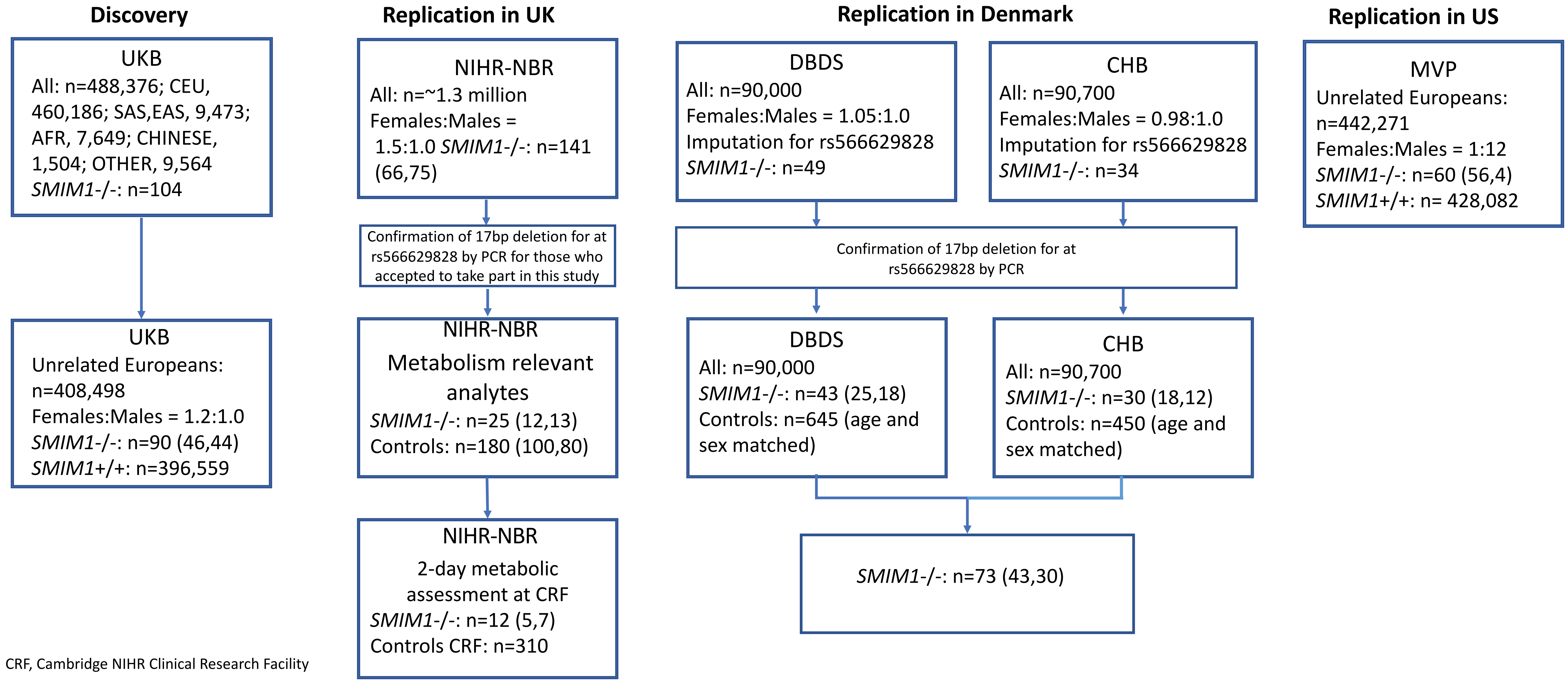

SF2 | Cohort information and the number of SMIM1-/- individuals per cohort

Genotype and phenotype data from four cohorts were used for the study. From left to right UK Biobank (UKB), NIHR-NBR, Danish Blood Donor Study (DBDS and The Copenhagen Hospital Biobank (CHB). The top row provides the number of participants for whom genotype (Vel phenotype in case of NIHR-NBR cohort) information was available and the female:male ratio. For the UKB the ethnicity of the participants is also provided (data taken from Bycroft et al., 2018)10. The middle row provides the number of SMIM1-/- individuals per cohort which were included in the study; between brackets (female/male) individuals.
