# Supplementary material for: SMIM1 absence is associated with reduced energy expenditure and excess weight
Source: Med. Author manuscript; Available in PMC 2025 Feb 24. (PMC7617389; doi:10.1016/j.medj.2024.05.015)
Supplement: Supplemental information [file EMS202150-supplement-Supplemental_information.zip › DataS1/SF files/SF1.pdf]

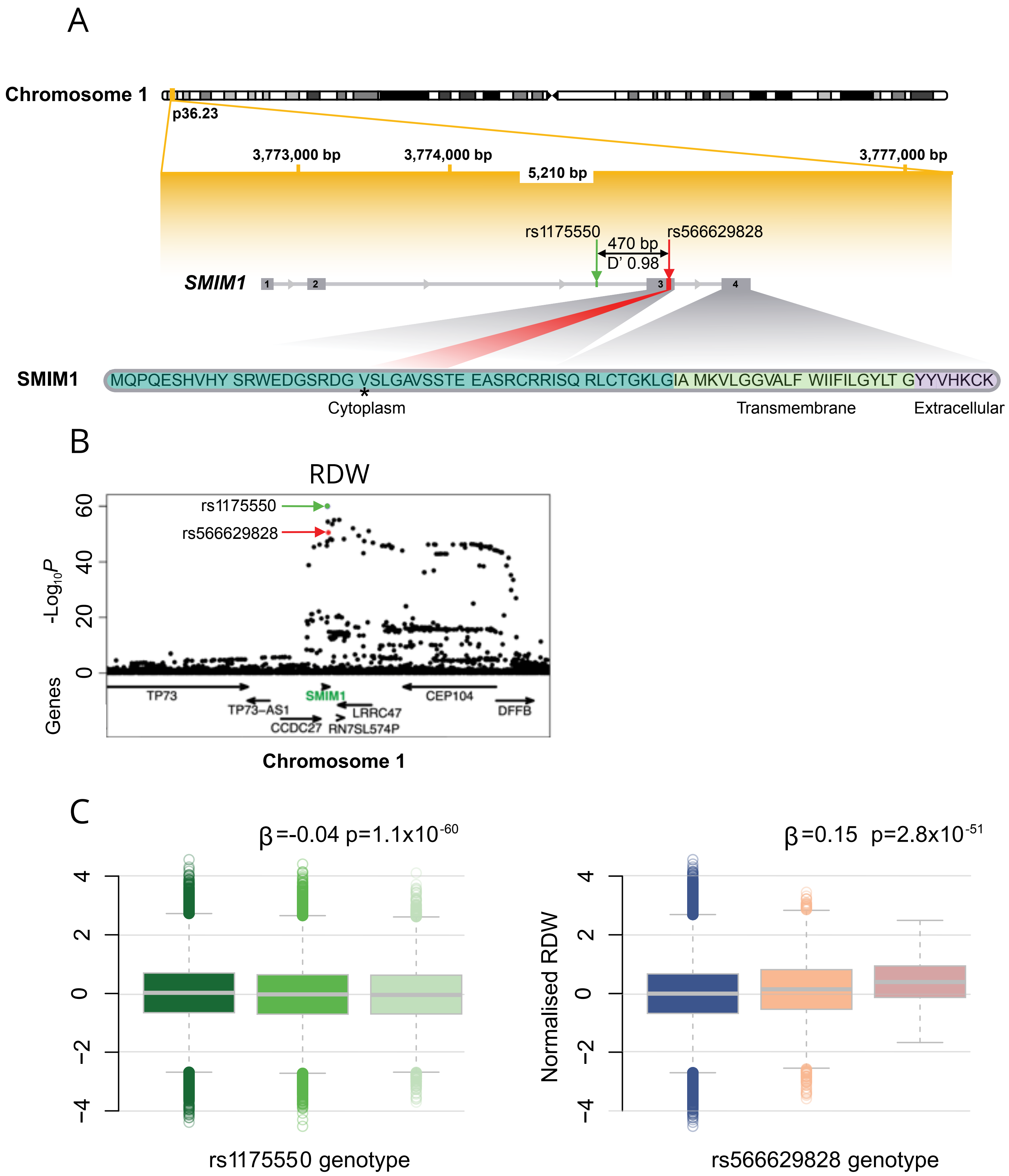

SF1 | Associations with red blood cell distribution width at the SMIM1 locus. (A) Upper panel: SMIM1 is localized on chromosome 1 at Chr1:p36.23. Middle panel: The genomic region harboring the SMIM1 gene between coordinates chr1:3,772,749-3,775,982 with the four grey boxes representing the SMIM1 exons, the grey lines being the introns and the arrows in the introns giving the transcript direction. The bright green and red vertical arrows indicate the position of the eQTL variant rs1175550 with an A and G nucleotide being reference and alternate, respectively and of the deletion variant rs566629828 with a reference containing the 17-bp sequence of GTCAGCCTAGGGGCTGT and the alternate lacking this sequence, respectively. The distance in base pairs (bp) and the D' value between the two variants are presented above and below the bidirectional horizontal arrow. Lower panel: The predicted sequence of the 78-amino acid (in single letter code) type II SMIM1 protein, with an estimated seven amino acids for the extracellular domain (purple), 23-amino acids for the transmembrane domain (light green) and with the remaining 48-amino acids being cytoplasmic (dark green). The asterisk indicates, on the protein, where the frame changes because of the deletion. **(B)** Locus zoom plots for SMIM1 (highlighted in green) genomic region showing the 100,000 bp centered on variant rs566629828; the normalized -Log10 P-values for the association between variants and red cell distribution width (RDW) are on the y-axis. The sentinel variant rs1175550 (eQTL variant in blood) is in green; after conditional analysis, it was observed that the 17-bp deletion (rs566629828, indicated in red) was independent of the sentinel variant associated with RDW. **(C)** Effect of the SMIM1 rs1175550 eQTL variant and rs566629828 (17-bp deletion) genotypes onto normalized RDW. The two boxplots have RDW distribution (y-axis) by genotype (x-axis). rs1175550 has ref/ref (A/A) in the darkest green, ref/alt (A/G) in green and alt/alt (G/G) in the lightest of green; rs566629828 have SMIM1+/+ in blue, SMIM1+/- in orange and SMIM1-/- in red. The beta and P-values for the associations are given above the boxplots, note the opposing directionality of the beta values, being negative for the eQTL variant (in blood SMIM1+/+ transcript level in alt/alt > alt/ref > ref/ref) and being positive for the deletion variant. This is in keeping with the observation that lower levels of SMIM1 RNA are associated with higher RDW levels. It is also worthwhile noting that in red cells effects of the eQTL and the 17-bp deletion variants are also observed in heterozygous individuals - this is in sharp contrast with the effect of the 17-bp deletion on body weight (Fig.1A).
